# Herpes simplex virus type 2 implicated in a case of acute disseminated encephalomyelitis

**DOI:** 10.1099/acmi.0.001018.v3

**Published:** 2025-11-17

**Authors:** Stuart Booth, Igor Starinskij, Stuart Gallacher, Peter Garmany

**Affiliations:** 1 NHS Greater Glasgow and Clyde, Queen Elizabeth University Hospital, 1345 Govan Road, Glasgow G51 4TF, UK; 2 Medical Research Council–University of Glasgow Centre for Virus Research, Henry Wellcome Building, Garscube Campus, 464 Bearsden Road, Glasgow G61 1QH, UK; 3 West of Scotland Specialist Virology Centre, New Lister Building, 8-16 Alexandra Parade, Glasgow G31 2ER, UK

**Keywords:** acute disseminated encephalomyelitis (ADEM), herpes simplex virus type 2 (HSV-2)

## Abstract

**Introduction.** Acute disseminated encephalomyelitis (ADEM) is a well-described neurological disorder that follows acute infection, vaccination and organ transplantation. It is characterized by sudden and widespread areas of inflammation in the central nervous system. Previous case reports have described ADEM with evidence of either recent or current herpes simplex virus type 1 infection. However, here, we report a rare, to our knowledge never before documented, case of ADEM associated with herpes simplex virus type 2 (HSV-2).

**Case report.** A 20-year-old man presented with weakness and sensory disturbance to the lower limbs, which had gradually progressed over the preceding 7 days, with associated fever, urinary retention and bowel incontinence. Magnetic resonance imaging was in keeping with a diagnosis of ADEM with mainly spinal involvement. Lumbar puncture revealed lymphocytic pleocytosis with elevated protein, and PCR was strongly positive for HSV-2. He was treated with aciclovir and dexamethasone, along with broad-spectrum antibiotics until negative bacterial and mycobacterial culture results were obtained. His functional status improved over the following months, but, despite prolonged rehabilitation, neurological sequelae remain.

**Conclusion.** HSV-2 may be considered a possible aetiological agent in cases of ADEM.

## Data Summary

Data for cerebrospinal fluid multiplex PCR result comparison were extracted from the laboratory information management system using Dedalus Telepath version 1.9 by a data manager independent from the project. The following parameters were retrieved: patient demographic information, location at time of request, sample collection date, result authorization date, sample type, test interpretation results and cycle threshold values. The data were exported in XLSX format for processing and analysis. Samples obtained from the specific patient described in the report, as well as samples that did not complete testing, were excluded. The final dataset comprised 3,270 samples collected between 27 February 2019 and 30 October 2021. All information except for specimen type, collection date and testing results was removed to preserve patient confidentiality.

## Introduction

Acute disseminated encephalomyelitis (ADEM) is a well-described neurological disorder that can complicate acute infection, vaccination and organ transplantation and often leads to acute urinary retention [1,7]. Preceding infection is not part of a formal definition of ADEM, which is considered an immune-mediated condition, although it is almost certainly one of the major triggers [8].

There are case reports of ADEM with evidence of recent or current herpes simplex virus (HSV) type 1 (HSV-1) infection as well as a survey reporting HSV genome detection in cerebrospinal fluid (CSF) in one out of three ADEM cases [9,12]. In contrast, there is no literature, to the best of the authors’ knowledge, describing an association between herpes simplex virus type 2 (HSV-2) and presentations of ADEM.

## Case presentation

A 20-year-old man presented to the emergency department of a large inner city teaching hospital complaining of weakness and sensory disturbance affecting his lower limbs which had gradually progressed over the preceding 7 days.

The patient described initial altered sensation in his left foot with associated limp which, over a period of 4 days, developed to painful paraesthesia of his entire left leg. On the morning of attending hospital, he reported a complete loss of sensation to both legs and an inability to stand or walk. At this time, there was associated urinary incontinence. He required being carried into the emergency department due to bilateral limb weakness.

He was fit and well prior to this, except for a single episode of pharyngitis and tonsillar exudate 14 days preceding initial neurological symptoms, which fully resolved with 7 days of oral phenoxymethylpenicillin (500 mg four times daily).

There was no history of recent travel or unwell contacts. He had been immunized fully in line with the standard UK vaccination schedule including coronavirus disease 2019 vaccination. There was no family history of inherited conditions including no previous familial neurological condition.

He had one female sexual partner of around 2 months, was heterosexual and reported no unprotected sex. There was no evidence of genital blistering or ulceration, and he did not report any genitourinary symptoms.

On examination, he was noted to have Medical Research Council (MRC) grade 0/5 power in both lower limbs, with complete sensory loss (pin prick, light touch, vibration and proprioception) from the T10 through S5 dermatomes. Both legs were areflexic with flaccid muscle tone. He was found to be in urinary retention, requiring urinary catheterization and was incontinent of faeces. He was febrile with a temperature of 38.8 °C, and observations were otherwise within normal parameters.

Computed tomography head scanning was performed with intravenous contrast and was unremarkable. Blood laboratory results, including full blood count, C-reactive protein, liver function and renal function, were essentially within normal limits, while CSF results can be found in Table 1, although viral PCR results were not available at this point.

**Table 1.** Trend of CSF results over the course of in-hospital treatment

Parameter	Reference range	Day 1 of treatment	Day 12 of treatment	Day 26 of treatment
Opening pressure	10–20 cmH_2_O	20 cmH_2_O	*Not obtained*	15 cmH_2_O
Appearance	Clear colourless fluid	Straw-coloured fluid	Straw-coloured fluid	Clear colourless fluid
Red cell count	<5/mm^3^	597/mm^3^	1,360/mm^3^	<5/mm^3^
White cell count	<5/mm^3^	425/mm^3^	41/mm^3^	10/mm^3^
Polymorphs	*Not applicable*	20%	10%	*Not specified*
Lymphocytes	*Not applicable*	80%	90%	*Not specified*
Protein	0.1–0.5 g l^−1^	3.3 g l^−1^	0.71 g l^−1^	0.46 g l^−1^
Glucose	2.5–4.5 mmol l^−1^	1.2 mmol l^−1^	1.7 mmol l^−1^	2.7 mmol l^−1^
HSV-2 PCR (Ct value)	*Not applicable*	Detected (22.2)	Detected (33.5)	Not detected
HSV-1, VZV, enterovirus, parechovirus PCR	*Not applicable*	Not detected	Not detected	Not detected

Empirical treatment was started (once daily unless stated otherwise):

- intravenous ceftriaxone 2 g twice daily,
- intravenous aciclovir 10 mg kg^−1^ three times daily,
- oral dexamethasone 10 mg twice daily,
- oral rifampicin 720 mg,
- oral isoniazid 300 mg,
- oral pyrazinamide 1,800 mg,
- oral ethambutol 1,200 mg,
- oral pyridoxine 10 mg.

At this point, the working diagnosis was a central nervous system (CNS) infection, such as CNS tuberculosis, but other common bacterial and viral pathogens were also covered with the empirical regimen.

At day 10 of admission, he reported new paraesthesia to both feet which progressed over the following 2 days to involve his entire lower limbs. On day 12, he was noted to have light touch and pinprick sensation to the soles of both feet. On day 13, it was noted that motor function was recovering with hip adduction power bilaterally at MRC grade 1/5.

Direct Gram staining of CSF was negative, and bacterial culture was performed with no growth of bacterial pathogens by 48-h incubation. Bacterial PCR was negative for *Haemophilus influenzae*, *Neisseria meningitidis* and *Streptococcus pneumoniae*. A total of 10 ml of CSF was sent for mycobacterial culture with no growth at 42-day incubation. The CSF samples underwent PCR testing using an in-house assay for HSV-1, HSV-2, varicella zoster virus, enteroviruses and parechoviruses. HSV-2 was detected in the first CSF sample at a cycle threshold (Ct) of 22.2. The patient was extensively investigated for infectious and autoimmune conditions, and the results can be found in Table 2.

**Table 2.** Summary of specialist testing results by local and reference laboratories All tests performed on samples collected during admission, except where specified otherwise for HSV serology.

Parameter	Sample type	Result (reference range, if applicable)
16s rDNA real-time PCR	CSF	No rDNA detected
*Mycobacterium tuberculosis* PCR	CSF	Negative
*N*-Methyl-d-aspartate receptor antibodies	CSF	Negative
Oligoclonal bands	Paired CSF and serum	Normal
Antineutrophil cytoplasmic antibodies	Serum	Negative
Antinuclear antibodies	Serum	Negative
Anti-streptolysin O titre	Serum	99 U ml^−1^ (<200 U ml^−1^)
Aquaporin4 antibodies	Serum	Negative
*Borrelia burgdorferi* IgM and IgG	Serum	Negative
Ganglioside screen*	Serum	Negative
Neuronal antibodies†	Serum	Negative
Myelin oligodendrocyte glycoprotein antibodies	Serum	Negative
Myeloperoxidase antibodies	Serum	<0.2 IU ml^−1^ (<3.5 IU ml^−1^)
Proteinase-3 antibodies	Serum	<0.2 IU ml^−1^ (<2.0 IU ml^−1^)
Hepatitis B surface antigen	Plasma	Negative
Hepatitis C antibodies	Plasma	Negative
HIV antibodies/antigen	Plasma	Negative
HSV total antibodies during admission	Plasma	Negative
HSV total IgM during admission	Plasma	Negative
HSV-1 IgG during admission	Plasma	Negative
HSV-1 IgG 7 months post-presentation	Serum	Negative
HSV-2 IgG during admission	Plasma	Negative
HSV-2 IgG 7 months post-presentation	Serum	Positive
Rapid plasma reagin	Plasma	Negative
*Treponema pallidum* antibodies	Plasma	Negative
*Treponema pallidum* particle agglutination assay	Plasma	Negative

*Ganglioside screen includes GM1 IgG, GM2 IgG, GD1a IgG, GD1b IgG, GQ1b IgG, GM1 IgM, GM2 IgM, GD1a IgM, GD1b IgM and GQ1b IgM.

†Neuronal antibodies include anti-HU, anti-Yo, anti-RI, anti-CV2, anti-MA2TA, anti-amphiphysin, anti-SOX1, anti-recoverin and anti-titin.

IgG, immunoglobulin G; IgM, immunoglobulin M; rDNA, ribosomal deoxyribonucleic acid.

**Fig. 1.**
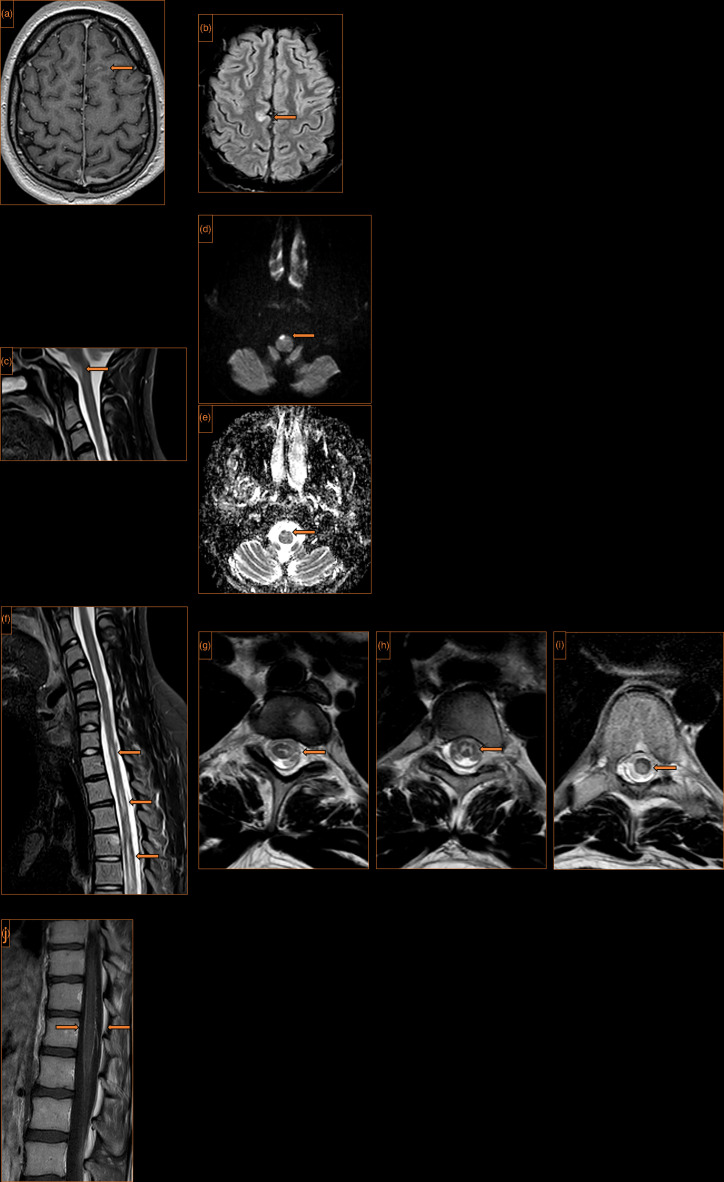
MRI of the head and spine. (**a**) Axial T1 post-gadolinium contrast of the brain. Focal leptomeningeal enhancement high left frontal lobe (arrow). (**b**) Axial T2 fluid-attenuated inversion recovery of the brain. A focal high signal lesion at the parafalcine cortex right frontal lobe (arrow). (**c**) Sagittal T2-weighted images of the craniocervical junction. A high signal lesion in the anterior medulla oblongata (arrow). (**d**) Axial diffusion-weighted imaging of the craniocervical junction. Restricted diffusion within the lesion (arrow). (**e**) Complementary low signal on the apparent diffusion coefficient map (arrow). (**f**) Sagittal T2-weighted images of the cervicothoracic spine. Multiple focal high signal lesions within the spinal cord, measuring up to two vertebral levels in length (arrows). These lesions are associated with cord swelling and did not enhance after contrast. (**g−i**) Axial T2-weighted images at three levels of the dorsal spine. Heterogeneity of location with the cord substance, with some predilection for grey matter (arrows). (**j**) Sagittal T1 of the lumbar spine with intravenous gadolinium contrast. Florid leptomeningeal enhancement over the surface of the conus (arrows).

Electromyography showed mild abnormalities in lower limb motor studies with preserved sensory studies but no clear motor activity in the lower limbs in keeping with a central pathology. T1, T2, diffusion weighted, short tau inversion recovery magnetic resonance imaging (MRI) of the head and spine with gadolinium enhancement was performed with findings largely confined to within the brain stem and spinal cord. There were multiple intramedullary cord lesions. The appearances were in keeping with a diagnosis of ADEM (Fig. 1). Predominant spinal involvement and enhancement of the spinal meninges are uncommon features but previously described [13,14].

A repeat lumbar puncture was performed on day 26 of admission, prior to completion of intravenous antiviral therapy. CSF testing results across the admission are summarized in Table 1. CSF microscopy with Gram staining, bacterial culture and viral PCR screen, excluding HSV-2, remained negative throughout.

MRI at this time showed interval improvement in intracranial and spinal appearances, with no new evidence of leptomeningeal enhancement and previous findings less appreciable in keeping with response to treatment.

Treatment was rationalized as culture results became available, with tuberculosis treatment continuing until negative mycobacterial culture. Intravenous aciclovir was continued for a total of 28 days before being switched to 500 mg valaciclovir once daily for a total of 6 months. Dexamethasone was discontinued at day 10 after liaison with the clinical neurology service.

He underwent extensive physiotherapy and had a gradual progressive improvement in motor and sensory function. On day 90 of admission, the patient was transferred to the physical disability rehabilitation unit (PDRU). At this time, he had MRC grade 2/5 bilateral lower limb weakness with normal sensation to pinprick and light touch, although he remained areflexic with flaccid muscle tone in T10-S5 myotomes. He remained catheterized due to multiple failed trials without catheter and was intermittently incontinent of faeces.

The patient underwent a further 45 days of physical rehabilitation in the PDRU. By the time of discharge, power had improved to MRC grade 4/5 on his right and 3/5 on his left lower limb. He was mobilizing with a wheelchair and was independent with all transfers and self-care. Bowel function normalized; however, the patient continued to require intermittent self-urinary catheterization 2–3 times weekly due to incomplete bladder emptying.

The patient made continued improvement over the next 3 years. His leg power had progressed to MRC grade 5/5 on the right and 4+/5 on the left and he had become independently mobile with elbow crutches. He was able to drive, work in retail for around 5 h per day and attend a gym. However, neurological sequelae, in the form of lower limb spasticity and chronic urinary retention, remain.

## Discussion

This is a case of ADEM with strong molecular and serological evidence of a concurrent HSV-2 CNS infection.

Acute haemorrhagic leucoencephalitis is the most severe and rapidly progressive form of ADEM [15,16]. Nonetheless, the absence of evidence of cerebral bleeding in this patient and non-hyperacute course both make this diagnosis unlikely.

CSF PCR was used as the primary aetiological diagnostic tool during the acute phase of illness. PCR does not directly measure the number of replicating virions, but the Ct values obtained using this method correlate with both disease severity and duration of illness [17,18]. To put the results into context, data from CSF PCR assays performed in the local virology laboratory over a 20-month period between 2019 and 2021 were reviewed (Table 3). In this case, the Ct of 22.2 is lower than ever obtained for any pathogen in the examined timeframe, compatible with an early phase of a severe infection.

**Table 3.** A review of 3,270 CSF PCR results using the same assay as the case

Analyte	No. of positive samples	Mean Ct (range)
HSV-1	20	31.1 (22.8–37.3)
HSV-2	13	30.6 (25.9–36.3)
Varicella zoster virus	26	31.2 (27.0–36.9)
Enterovirus	23	33.9 (29.9–39.5)

A few alternative explanations should be considered. Firstly, HSV-2 encephalitis, although uncommon in general, is in keeping with the finding of a high amount of HSV-2 DNA in the CSF [19]. On the other hand, although the neurological features of both ADEM and HSV encephalitis can be diverse, there were no clinical signs of generalized cerebral inflammation, such as seizures and reduced consciousness level. In addition, widespread MRI findings were in keeping with ADEM rather than HSV-2 encephalitis, which is often confined to the temporal and frontal lobes [4,19]. Secondly, postinfectious immune-mediated encephalitis is often described as one of the forms of ADEM but can also present differently [5,20]. It can be distinguished from ADEM by an absence of new lesions compared to the areas affected by HSV during the first episode of overt encephalitis with negative viral PCR during the second presentation [20]. Here, there was no preceding illness suggestive of HSV-2 encephalitis and the viral PCR is positive. Finally, acute HSV-2 CNS infection could have coincided with ADEM with distinct aetiology. In this case, pharyngitis antecedent to neurological presentation was indeed diagnosed and theoretically could have been the original cause of autoimmune neurological disease. However, its causative agent had never been identified; hence, it is impossible to conclusively link this presentation to the past event.

Multiple sclerosis (MS), the first attack of which can be highly similar to ADEM, should be thought of when evaluating the long-term prognosis of a patient with ADEM [21,22]. All three proposed differentiation criteria (atypical sign – paraplegia, absence of oligoclonal bands and grey matter involvement) present in this case were supportive of ADEM rather than MS [23]. To date, our patient had not experienced recurrent or new symptoms suggestive of MS.

One limitation to the primary diagnosis is that a biopsy with relevant histological investigations is required for a final confirmation of ADEM. However, a close temporal association, robust detection by repeated PCR assays as well as the known role of a closely related HSV-1 in ADEM pathogenesis strongly support HSV-2 as the neurological disease-causing agent here. Additionally, we demonstrated HSV-2 type-specific immunoglobulin G seroconversion (Table 2), further solidifying the case for an acute HSV-2 CNS infection, while imaging was most in keeping with ADEM rather than alternative neurological conditions.

In summary, we describe a case of ADEM affecting primarily the spinal cord with strong molecular and serological evidence of HSV-2 CNS infection. The case was treated with a variety of antimicrobials, notably aciclovir, and a 10-day course of dexamethasone. The patient had a slow but steady improvement in neurological function over a period of months, but years later, neurological sequelae remain.

## Abbreviations

ADEM: acute disseminated encephalomyelitis
CNS: central nervous system
CSF: cerebrospinal fluid
Ct: cycle threshold
D: detected
DL: detected at low level
Ent: Enterovirus
HSV-1: herpes simplex virus type 1
HSV-2: herpes simplex virus type 2
HSV: herpes simplex virus
I: not tested as sample insufficient
MRC: Medical Research Council
MRI: magnetic resonance imaging
MS: multiple sclerosis
N: not detected
PCR: polymerase chain reaction
PDRU: physical disability rehabilitation unit
VZV: Varicella Zoster virus
